# Supplementary material for: Dropping diversity of products of large US firms: Models and measures
Source: PLoS One. 2022 Mar 16;17(3):e0264330. doi: 10.1371/journal.pone.0264330 (PMC8926282; doi:10.1371/journal.pone.0264330)
Supplement: S1 File — (PDF) [file pone.0264330.s001.pdf]

Supporting information

S1 Appendix. Definition of Industry Specificity.

Let $F = \{f_1, f_2, ..., f_n\}$ be the set of firms (semantic vectors) that exist in some particular year, and let each industry in a reference classification, C, be a set of firms. So, a reference classification is a set of industries (i.e., classes of firms): $C = \{c_1, c_2, ..., c_m\} = \{\{f_i, ..., f_l\}, \{f_j, ..., f_m\}, ..., \{f_k, ..., f_n\}\}$. Here, we let the set of firms in each 4-digit SIC Code to be our reference classification. In this case, C partitions the set, F, of all firms (that exist in a given year), so that every firm is in exactly one class (SIC Code). Then, we can can write the *mean between-class similarity* of a firm similarity matrix, M, for a given year Y as the mean similarity between pairs of firms which do not belong to the same industry, averaged over all industries:

$$\sum_{i=1}^{|C|} \left(\frac{\sum_{f \in c_i, f' \in c_j, i \neq j} \text{sim}_M(f, f')}{|c_i| \times (|c_i| - 1)} \right) \frac{1}{|C|} \tag{1}$$

To improve readability we typically leave the year Y implicit.

We can also write the mean *mean within-class similarity* of a firm similarity matrix M in year Y as the mean similarity of all pairs of firms within the same industry, averaged over all industries:

$$\sum_{i=1}^{|C|} \left(\frac{\sum_{f \in c_i, f' \in c_i, f \neq f'} \text{sim}_M(f, f')}{|c_i| \times (|c_i| - 1)} \right) \frac{1}{|C|} \tag{2}$$

Finally, we define the *Industry Specificity (relative to the SIC)* of a firm similarity matrix M as its mean within-class similarity (according to M) divided by its mean between-class similarity; that is, the SIC Industry Specificity of M is:

$$\frac{\text{mean within SIC Code similarity of } M}{\text{mean between SIC Code similarity of } M} \tag{3}$$

The SIC industry specificity of model M is the mean similarity (according to M) of firms within each industry (according to the SIC) expressed in units of the mean global firm similarity.

S2 Appendix. Adjusting for Maximum Diversity. It might be useful to remove

the change in the number of species/industries of the ecosystem for the diversity measures that use similarity matrices.

Take the definition for diversity given by Equation 3 in the main text. Maximum diversity occurs when the similarity matrix is an identity matrix, $Z = I$ and the abundance vector ${}^{balanced}a$ is perfectly balanced such that $a_{y,1} = ... = a_{y,s} = \frac{1}{s}$. Then,

$$ {}^{q}D({}^{balanced}a, I) = (\sum_{i}^{s} a_i (\sum_{j}^{s} I_{ij} a_j)^{q-1})^{\frac{1}{1-q}} \tag{4} $$

$$ = (\sum_{i}^{s} a_i (a_i)^{q-1})^{\frac{1}{1-q}} \tag{5} $$

$$ = (\sum_{i}^{s} (\frac{1}{s})^{q})^{\frac{1}{1-q}} \tag{6} $$

$$ = s. \tag{7} $$

Therefore, the adjusted diversity of order q is given by

$$ {}^{q}D_{adj}(a, Z) = \frac{{}^{q}D(a, Z)}{s}. \tag{8} $$

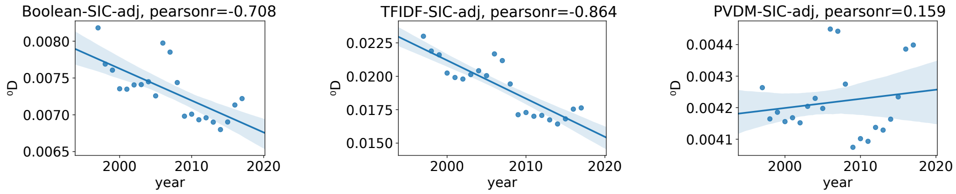

Fig 1. Annual economic diversity of order zero, ${}^{0}D$, adjusted for varying number of the 4-digit SIC Codes that are instantiated, according to models: Boolean (left), TF-IDF (middle), and PV-DM (right). Each model includes a scatter plot of diversities and a linear regression fit along with its 90% confidence interval.
